# Calculation of the Maximum Temperature of Diester-Based Magnetic Fluid Layers in High-Speed Seals

**DOI:** 10.3390/nano13061019

**Published:** 2023-03-11

**Authors:** Yanhong Cheng, Zhe Su, Jiayi Zhou, Zhifeng Liu, Decai Li, Caixia Zhang, Jingjing Xu

**Affiliations:** 1 Institute of Advanced Manufacturing and Intelligent Technology, Beijing University of Technology, Beijing 100124, China; chengyh@bjut.edu.cn (Y.C.); suzhe@emails.bjut.edu.cn (Z.S.); zhoujy@emails.bjut.edu.cn (J.Z.); xujj0908@bjut.edu.cn (J.X.); 2 Key Laboratory of Advanced Manufacturing and Intelligent Technology for High-End CNC Equipment, Changchun 130015, China; 3 State Key Laboratory of Tribology, Tsinghua University, Beijing 100084, China; lidecai@tsinghua.mail.edu.cn

**Keywords:** magnetic fluid, maximum temperature, high-speed seal, rheological properties, temperature calculation

## Abstract

Magnetic fluids, as smart nanomaterials, have been successfully used in sealing applications and other fields. However, the temperature of magnetic fluids in the sealing gap is a key factor affecting sealing performances, limiting their application in high-speed sealing fields. Since obtaining a direct measurement of the magnetic fluid’s temperature is difficult, due to the small clearance, accurately calculating the maximum temperature of the magnetic fluid layer in high-speed seals is crucial. Herein, a mathematical model for calculating the maximum temperature of the magnetic fluid layer was established, by using a reasonable simplification of high-speed sealing conditions, and the calculation formula was modified by studying the rheological properties of the diester-based magnetic fluid. The results suggest that the calculation of the maximum temperature is influenced by viscous dissipation, and both are related to the rheological characteristics of magnetic fluids. When the influence of rheological properties is ignored, the calculation results are not accurate for higher-velocity seals, but the calculation model applies to lower-velocity seals. When the influence of rheological properties is considered, the calculation results obtained by the corrected formula are more accurate, and they are applicable to both lower- and higher-velocity seals. This work can help us more accurately and conveniently estimate the maximum temperature of magnetic fluids in high-speed seal applications, which is of theoretical and practical research significance for determining sealing performances and thermal designs.

## 1. Introduction

A magnetic fluid (MF), a stable colloidal solution, is usually formed by ferromagnetic nanoparticles that are uniformly suspended in a nonmagnetic carrier fluid, utilizing a surfactant [1,2]. It can exhibit fluidity like any other traditional fluids and magnetic properties that are similar to other solid magnetic materials, in the presence of magnetic fields. Magnetic fluid is responsive to magnetic fields and can be controlled and manipulated by magnetic fields. Thanks to its special characteristics, magnetic fluid has found applications in various fields, such as sealing, damping, and sensing [3,4]. In a range of industrial applications, sealing is one of the most successful and widely used examples, and it mainly utilizes the magnetization characteristic of magnetic fluids. The magnetization property is not only related to the magnetic field’s strength but also affected by the temperature of magnetic fluids, determining the sealing performance and further application development.

In previous studies, a variety of factors affecting the performance of magnetic fluid seals have been extensively investigated. Studies on the performance of magnetic fluid low-speed seals occupy the majority of them [5,6,7]. The design and optimization of different sealing structures (pole piece, pole tooth, gap size, permanent magnet, etc.) are mainly studied, which will affect the magnetic field’s gradients in the sealing gap, resulting in different magnetic pressure gradients for achieving different pressure resistances. The mature application of magnetic fluid in the field of low-speed seals has been successfully achieved by conducting these research studies. However, there are few studies on the performance of magnetic fluid high-speed seals, and their applications are not yet mature. Some previous studies on two major influencing factors, centrifugal force and viscous dissipation, have shown that the temperature increase in magnetic fluids caused by viscous dissipation is the root cause of high-speed sealing failure [8,9,10]. In high-speed seals, viscous dissipation increases, and the temperature of the magnetic fluid rises, leading to a diminution in magnetization strength and an acceleration in carrier fluid evaporation; the pressure resistance and lifespan of sealing decrease.

Several studies have been conducted on viscous dissipation and temperature in magnetic fluid seals. As a non-contact seal, although the friction torque is generated by internal friction in the fluid, the heat accumulated during operations can be significant, due to the small gap, the low volume of magnetic fluids, and the high rotational speed. Hence, some research has focused on the two important parameters of torque friction and viscous dissipation, as they determine the temperature of magnetic fluids in the seal. Li presented analytical methods for studying the technical issues of magnetic fluid seals, such as high speeds and large diameters. It was observed that the primary heat source was viscous drag, and the viscosity of magnetic fluids changes with temperature, leading to complex heat dissipation [11]. Cai et al. theoretically analyzed the starting friction torque of revolving magnetic fluid seals, based on their microstructure and viscosity. The results explained the relationship between viscosity and the starting friction torque and showed that temperature is one of the main influencing factors [12]. Based on their research, Cheng et al. derived the viscous resistance torque of the magnetic fluid seal, experimentally measured the rheological properties of magnetic fluid and the starting resistance torque, and further explored the mechanism of the intrinsic influence of viscosity on the friction torque [13]. Szczech conducted a theoretical analysis and experimental studies on torque friction in magnetic fluid seals. The viscosity, under the effect of magnetic fields, was measured in the study, but the comparison of the results also revealed a large difference between the experimental and calculated values in some cases. Considering the influence of temperature on the viscosity of magnetic fluid at higher speed seals was proposed [14]. Hu et al. investigated the effects of the loss of torque, on magnetic fluid rotating-shaft seals. They found that the loss of torque is affected by factors such as the characteristics of magnetic fluid. The viscosity increased with the magnetic field’s strength within a certain range, and the increase in the loss of torque tended to slow down when the magnetic fluid was saturated [15]. Krakov et al. numerically studied the flow and convection of magnetic fluid seals, and they analyzed the influence of thermomagnetic convection on the temperature distribution in high-speed seals. It was shown that magnetic fluid is heated due to viscous friction, but the thermomagnetic convection levels of the temperature distribution lower the temperature of magnetic fluids in the gap [16]. Zhang et al. investigated the temperature of magnetic fluid lubricating films in a spiral groove mechanical seal, using the separation variable method. Their calculation procedure only considered the relationship between the magnetic fluid’s viscosity and strength, and temperature at the low-speed range was discussed [17].

In addition, some experimental studies on the temperature in the sealing gap have also been carried out. Szydlo et al. conducted an experimental study on the temperature and frictional moment of magnetic fluid seals operating under vacuum conditions. It was concluded that the temperature of the seal increases with an increase in rotational speed and the number of sealing stages, both resulting in high viscous drag [18]. Borbáth et al. measured the effect of rotational speed and viscosity on the magnetic fluid seal’s temperature and breakaway torque. They observed that the increase in rotational speed led to an increase in heat generated by viscous friction, resulting in a reduction in seal capacity. They also pointed out that the measurement does not reflect the exact temperature of the magnetic fluid itself but reflects the seal, and further experiments are needed to obtain more precise results [19]. Szczech et al. researched the effect of temperature on the pressure transfer mechanism of magnetic fluid seals. They supposed that, with the increase in temperature, the saturation magnetization, viscosity, and other properties of the magnetic fluid were influenced, causing seal leakage [20]. Vlasov et al. developed an experimental system to measure the effect of changes in structural and operational parameters during the friction moment and heating of magnetic fluid seals. The results showed that friction torque increased with speed, and heating varied nonlinearly with time, due to the nonlinear variation in viscosity occurring with the rotational speed and temperature [21]. Chen et al. studied the change rule of magnetic fluid temperatures and the influence on the sealing performance, by using simulations and experimental methods. The results showed that frictional heat had a significant effect on sealing capacity, and sealing capacity decreased with an increase in speed due to the temperature increase [22]. Mitamura et al. measured the magnetic fluid temperature in a miniature magnetic fluid seal installed in a rotary pump, and they investigated the cooling conditions of magnetic fluid seals that control the magnetic fluid temperature. It was observed that, the capacity of the magnetic fluid seal was not affected by magnetic fluid temperature increases due to viscous friction, when the heat transfer coefficient of the seal’s housing reaches a greater value [23].

In summary, previous studies conducted some theoretical and experimental studies on temperature and viscous dissipation in magnetic fluid seals. In terms of theoretical research, the effects of viscosity and other factors on friction torque and viscous heat were mostly studied, but the rheological properties of magnetic fluids under sealing conditions have rarely been systematically considered in viscous dissipation calculations. In terms of experimental research, most studies focused on the effect of temperature on the sealing capacity, but the temperature of magnetic fluids in the seal makes it difficult to achieve direct and accurate measurements, due to the narrow sealing gap. Therefore, the calculation, and quantification, of temperature in magnetic fluid seals is still incomplete, especially with respect to studying the maximum temperature of magnetic fluids in high-speed seals. This article establishes a calculation model of the maximum temperature of magnetic fluid layers in the sealing gap, by simplifying high-speed sealing conditions. The rheological properties of magnetic fluid under the simulation conditions of high-speed seals are measured, and the formula for calculating the viscous dissipation and MF temperature is subsequently modified. The maximum temperature of diester-based magnetic fluids is calculated accurately, under the condition of high-speed seals without cooling. This theoretical calculation can help to guide the application of magnetic fluids in high-speed sealing fields conveniently and quickly, such as judging the performance of magnetic fluids, sealing capacity, the cooling conditions of the seal, etc. We anticipate that our study can lay theoretical and practical foundations for promoting the research of magnetic fluid high-speed seals, which have great potential applications in military and aerospace and sealing other high-end equipment, in the future.

## 2. Materials and Methods

### 2.1. Materials

**Figure 1:**
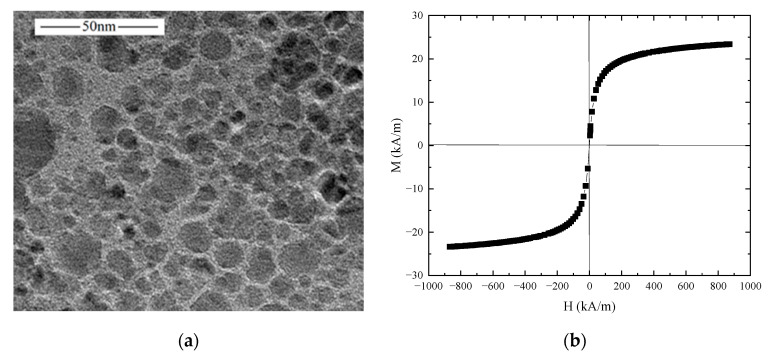
(**a**) TEM image of nanoparticles; (**b**) VSM magnetization curve for the magnetic fluid.

The Fe_3_O_4_-diester magnetic fluid studied herein was prepared via chemical co-precipitation by our group. The sample was successfully applied in commercial conditions and placed stably for more than three years without visible particle agglomeration. The TEM image of Fe_3_O_4_ nanoparticles was observed by the JEM-2100F transmission electron microscope. Figure 1a illustrates that the sample contains uniformly dispersed particles, with almost spherical shapes, and the average particle size is around 10 nm. For this size range, the particles can be stably suspended in the carrier fluid without sedimentation. The magnetization curve of the diester-based magnetic fluid was measured by a Lake Shore vibrating sample magnetometer (VSM) at room temperature, as shown in Figure 1b. Some physical parameters of the examined magnetic fluid are expressed in Table 1.

### 2.2. Model Formulation

**Figure 2:**
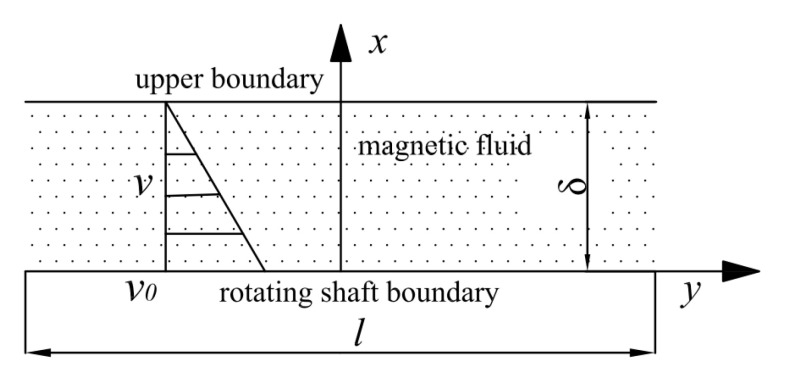
Two-dimensional model for temperature calculations.

In this study, the magnetic fluid’s flow in a high-speed seal clearance is simplified into an incompressible stable flow, in a two-dimensional plate model. In the actual working condition, the sealing gap is narrow, and the magnetic fluid is wrapped by the shaft, pole pieces, and shell; thus, the temperature and heat transfer process of magnetic fluids in the clearance cannot be measured directly. Therefore, the following reasonable assumptions and simplifications were introduced, to calculate the temperature of the magnetic fluid layer in high-speed seals: (1) completely axisymmetric; (2) ignoring the heat transfer along the axial direction, and only the radial heat transfer is considered; (3) the height of the magnetic fluid layer is far smaller than the radius of the shaft, so it can be considered as a flat infinite plate model; (4) the characteristic length of the fluid layer is much larger than its height, and the temperature of the magnetic fluid in the central layer is considered in engineering calculations. A schematic diagram of the calculation model herein is shown in Figure 2: for a viscous fluid heat conduction layer with a certain height, the upper boundary is immobile, and the lower boundary moves along the plane (corresponding to the velocity of the rotating shaft’s surface).

Here, $\rho_{ff}$ is the density of fluid; v is velocity; η is the absolute viscosity of the fluid; c and λ denote the specific heat at constant pressure and the thermal conductivity of the fluid, respectively; Φ is the viscous dissipation term; x is the axis that goes through the fluid layer.

The following governing equations are used to study the flow and heat transfer of magnetic fluid in the above-defined model.

(1) $$\rho_{ff}\frac{\partial v}{\partial \tau }=\eta \frac{\partial^{2}v}{\partial x^{2}}$$

(2) $$\rho_{ff}c\frac{\partial T}{\partial \tau }=\lambda \frac{\partial^{2}T}{\partial x^{2}}+\Phi$$

For a steady state, the velocity distribution at a certain fluid layer height can be approximately regarded as the following linear relationship:

(3) $$v=v_{0}\left(1-\frac{x}{\delta }\right)$$

where $v_{0}=\omega r$ represents the velocity of the rotating shaft’s surface, and δ represents the height of the fluid layer.

For a given rotating speed, viscous dissipation is determined when the viscosity of a fluid is regarded as a constant, and it can be calculated by the following formula.

(4) $$\Phi =\eta {\left(\frac{dv}{dx}\right)}^{2}=\eta \frac{v_{0}{}^{2}}{\delta^{2}}$$

Therefore, the energy in Equation (2) can be expressed as follows.

(5) $$\frac{\partial^{2}T}{\partial x^{2}}+\frac{\eta v_{0}{}^{2}}{\lambda \delta^{2}}=0$$

The following dimensionless parameters are introduced to transform the above Formula (5) into the dimensionless form:

(6) $$\hat{x}=\frac{x}{\delta }$$

(7) $$\hat{T}=\frac{T-T_{0}}{T_{0}}$$

(8) $$Br=\frac{\eta v_{0}{}^{2}}{\lambda T_{0}}$$

where $\hat{x}$ represents dimensionless coordinates, $\hat{T}$ represents dimensionless temperature, Br represents the Brinkman number, and $T_{0}$ represents boundary temperatures.

Substituting dimensionless parameters (6)–(8) into Formula (5), the dimensionless expression of the temperature field can be obtained.

(9) $$\frac{d^{2}\hat{T}}{d{\hat{x}}^{2}}+Br=0$$

By carrying out the double integration of Equation (9), the general solution of dimensionless temperature is obtained:

(10) $$\hat{T}=-\frac{Br}{2}{\hat{x}}^{2}+c_{1}\hat{x}+c_{2}$$

where $c_{1}$ and $c_{2}$ are constants.

### 2.3. Model Solving

Under the condition of high-speed sealing, it is considered that the heat flux generated by viscous dissipation is constant throughout the boundary between the rotating shaft’s surface and the magnetic fluid, and the upper boundary between the magnetic fluid and the pole piece surface maintains a constant temperature. Therefore, the following boundary conditions were applied to solve Equation (10):

(11) $$\frac{d\hat{T}}{d\hat{x}}{/}_{\hat{x}=0}={\hat{q}}_{0},\hat{T}{/}_{\hat{x}=1}=0$$

where ${\hat{q}}_{0}$ is the dimensionless heat flux, and the relationship between the dimensionless heat flux and heat flux is described as follows:

(12) $${\hat{q}}_{0}=-\frac{\delta }{\lambda T_{0}}q_{0}$$

By substituting boundary condition (11) into Equation (10), the following integral constants can be obtained:

(13) $$c_{1}={\hat{q}}_{0}$$

(14) $$c_{2}=\frac{Br}{2}-{\hat{q}}_{0}$$

The dimensionless temperature distribution expression of the magnetic fluid layer is further obtained:

(15) $$\hat{T}=\frac{Br}{2}\left[1-{\hat{x}}^{2}-\frac{2{\hat{q}}_{0}}{Br}\left(1-\hat{x}\right)\right]$$

According to dimensionless parameter expressions (6)–(8), the dimensional expression of the magnetic fluid’s temperature distribution is as follows:

(16) $$T\left(x\right)=T_{0}+\frac{\eta v_{0}{}^{2}}{2\lambda }\left(1-\frac{x^{2}}{\delta^{2}}\right)+\frac{q_{0}\delta }{\lambda }\left(1-\frac{x}{\delta }\right)$$

We take a derivative of Formula (15) and obtain the maximum dimensionless temperature when $d\hat{T}/d\hat{x}=0$; then, the following is obtained:

(17) $$\hat{x}=\frac{{\hat{q}}_{0}}{Br}$$

We further obtain the following equation:

(18) $$x=\frac{-\delta^{2}q_{0}}{\eta v_{0}{}^{2}}$$

By substituting the above Formula (18) into Formula (16), the maximum temperature of the magnetic fluid layer in the high-speed seal can be calculated as follows:

(19) $$T_{\max}=T_{0}+\frac{\eta v_{0}{}^{2}}{2\lambda }{\left(1+\frac{q_{0}\delta }{\eta v_{0}{}^{2}}\right)}^{2}$$

### 2.4. Rheological Properties of MF

**Figure 3:**
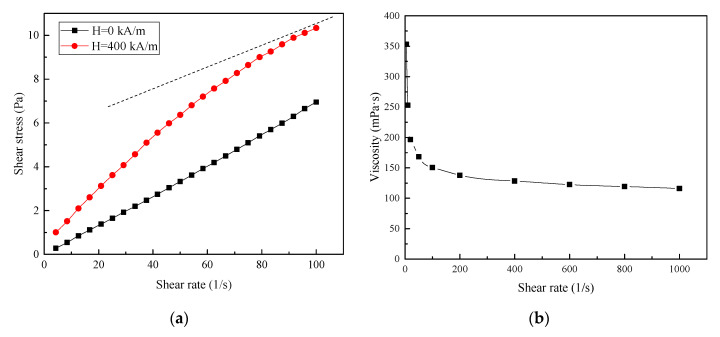
(**a**) Shear stress versus the shear rate of diester-based magnetic fluids at magnetic fields of 0 kA/m and 400 kA/m; (**b**) viscosity versus the shear rate at 400 kA/m.

Viscosity is one of the important physical parameters of MF, and it is affected by magnetic fields, shear rate, and temperature. To accurately obtain the viscosity of MF under the sealing conditions, an Anton Paar MCR302 rotational rheometer was used, to measure the rheological properties of MF in a simulated seal environment. The relationship between the shear stress and shear rate was measured at room temperature, as shown in Figure 3a. It can be observed that shear stress increases linearly with the shear rate in the absence of magnetic fields, reflecting Newtonian flow characteristics. Shear stress increases nonlinearly with the shear rate in the presence of magnetic fields, exhibiting the non-Newtonian flow behavior of shear thinning. However, magnetic fluid cannot be diluted infinitely, and will gradually converge to its asymptote under the action of the shear rate, as shown in the dashed line of Figure 3a. The slope of this asymptotic line reflects the limiting viscosity coefficient of the magnetic fluid. The viscosity at different shear rates was further measured for a magnetic field of 400 kA/m, as shown in Figure 3b. Viscosity decreases significantly with increasing shear rates at low shear rate ranges, but viscosity changes tend to level off and gradually approach the ultimate shear viscosity at a higher shear rate range, which is consistent with the results shown in Figure 3a. For a high-speed magnetic fluid seal, the shear rate is large enough (>1000 s^−1^) to allow the viscosity to reach high shear-limiting viscosities.

**Figure 4:**
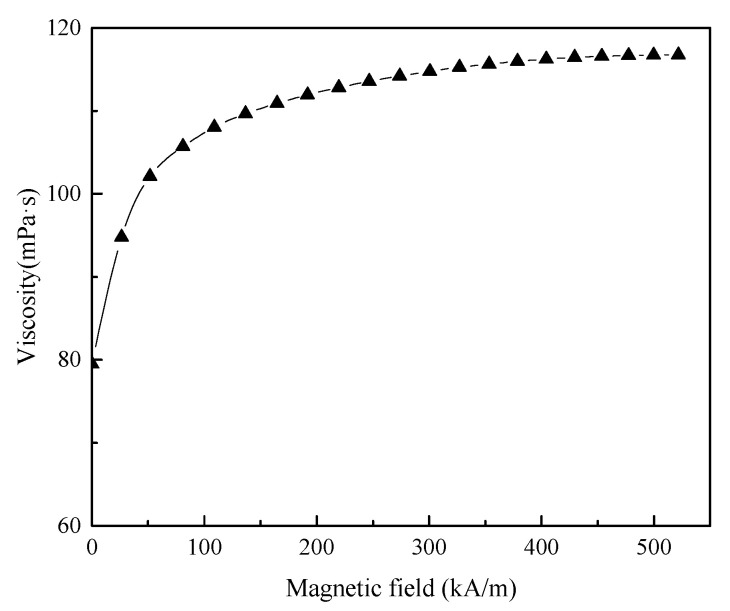
Relationship between the viscosity of the magnetic fluid and the magnetic field strength.

Magnetic fluids produce magnetic viscous effects under a magnetic field, and the variation in viscosity with respect to the magnetic field was measured experimentally at a shear rate of 1000 s^−1^, as indicated in Figure 4. Viscosity initially increased significantly with the enhancement of the magnetic field; then, viscosity no longer changed significantly and reached the approximate saturation state when the magnetic field was strong enough (>400 kA/m). The average magnetic field strength within the gap of a high-speed magnetic fluid seal, is usually greater than 400 kA/m; thus, viscosity is deemed to have reached magnetic saturation viscosity.

**Figure 5:**
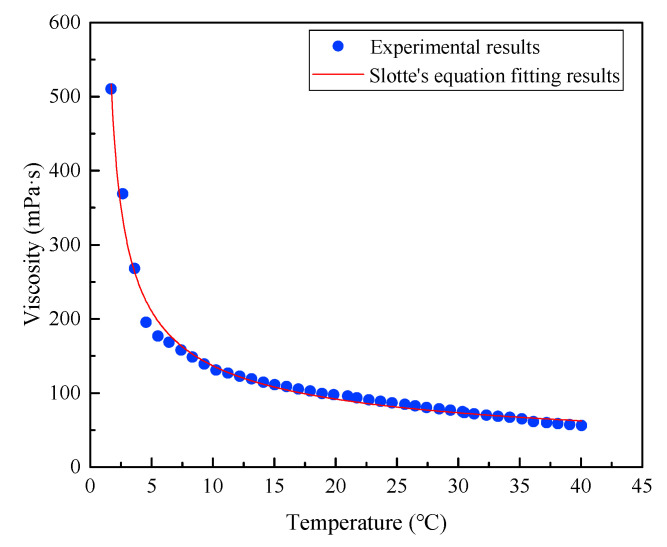
Viscosity–temperature curve of magnetic fluid.

Based on the results of Figure 3 and Figure 4, it is clear that the viscosity of the magnetic fluid achieved magnetic saturation and the ultimate shear state under high-speed sealing conditions, due to the sufficiently strong magnetic field and high shear rate. Therefore, the temperature is the key factor affecting the variation in viscosity under high-speed sealing conditions, ignoring the influence of the magnetic field and the shear rate. The viscosity–temperature curve of diester-based magnetic fluids was experimentally measured at a magnetic field strength of 400 kA/m and a shear rate of 1000 s^−1^. As depicted in Figure 5, viscosity gradually decreases with an increase in temperature, showing a nonlinear relationship, and the decrement becomes slower at higher temperatures.

### 2.5. Calculation of Viscous Frictional Dissipation for High-Speed Magnetic Fluid Seal

Viscosity is the root cause of thermal dissipation within the magnetic fluid seal, and the temperature increase generated by viscous frictional dissipation is the key reason for the degradation, or even failure, of high-speed magnetic fluid seals. At the starting operation of high-speed seals, the magnetic fluid’s temperature was lower and its viscosity was greater, resulting in higher dissipation power due to viscous friction, which caused the temperature increase in the seal gap. When magnetic fluid temperature increases, viscosity decreases and the dissipation power decreases. Therefore, viscosity, dissipation power, and temperature interact and influence each other. In order to accurately calculate the thermal dissipation power during high-speed seal operations, the viscosity–temperature relationship of magnetic fluids is taken into account. Based on the study of rheological performances in Section 2.4, the viscous frictional dissipation is derived and modified as follows.

The basic form of magnetohydrodynamic equations is similar to that of conventional fluids, with the exception that magnetic fluid is subjected to an additional magnetic force in the presence of magnetic fields. In high-speed magnetic fluid seal conditions, the following assumptions are made: (1) Magnetic fluid is a homogeneous incompressible Newtonian fluid; (2) it achieved a stable state, ∂()/∂t=0; (3) the sealing structure is completely axisymmetric, ∂()/∂θ=0; (4) radial velocity and axial velocity are neglected, $v_{r}=0$, $v_{z}=0$; (5) gravity is neglected. Based on the governing equations of magnetohydrodynamics in a cylindrical coordinate system, the equations are simplified as:

(20) $$\frac{\partial v_{r}}{\partial r}+\frac{\partial v_{\theta }}{r\partial \theta }+\frac{\partial v_{z}}{\partial z}+\frac{v_{r}}{r}=0$$

(21) $$\rho_{ff}\frac{v_{\theta }^{2}}{r}=\frac{\partial p^{*}}{\partial r}$$

(22) $$\frac{\partial^{2}v_{\theta }}{\partial r^{2}}+\frac{1}{r}\frac{\partial v_{\theta }}{\partial r}-\frac{v_{\theta }}{r^{2}}=0$$

(23) $$\frac{\partial p^{*}}{\partial z}=0$$

where $p^{*}=p-\int_{0}^{H}\mu_{0}MdH$. The boundary conditions are given as follows:

(24) $$r=r_{1},\;v_{\theta }=r_{1}\omega_{};\;r=r_{2},\;v_{\theta }=0$$

where $r_{1}$ is the radius of the shaft, $r_{2}$ is the radius of the pole piece, and ω is the angular velocity of the shaft.

By integrating Equation (22), and then substituting the boundary conditions in (24), the analytical solution of the tangential velocity distribution in the θ direction is obtained as follows:

(25) $$\;v_{\theta }=\frac{\omega r_{1}^{2}r_{2}^{2}}{r_{2}^{2}-r_{1}^{2}}\cdot \frac{1}{r}-\frac{\omega r_{1}^{2}}{r_{2}^{2}-r_{1}^{2}}\cdot r=\alpha \frac{1}{r}+\beta r$$

where $\alpha =\frac{\omega r_{1}^{2}r_{2}^{2}}{r_{2}^{2}-r_{1}^{2}}$ and $\beta =-\frac{\omega r_{1}^{2}}{r_{2}^{2}-r_{1}^{2}}$.

The subjected viscous shear stress of the magnetic fluid in the sealing gap is expressed as follows:

(26) $$\tau_{r,\theta }=\eta \left(\frac{\partial V_{\theta }}{\partial r}+\frac{\partial v_{r}}{r\partial \theta }-\frac{V_{\theta }}{r}\right)$$

where η is the viscosity of the magnetic fluid. By substituting velocity expression (25) into Equation (26), the viscous resistance torque acting upon the cylindrical surface of the magnetic fluid high-speed seal’s spindle is obtained, according to the viscous shear force:

(27) $$\Gamma =2\pi r_{1}\cdot l\cdot \tau_{r,\theta }\cdot r_{1}=4\pi l\eta \frac{\omega r_{1}^{2}r_{2}^{2}}{r_{2}^{2}-r_{1}^{2}}$$

where *l* indicates the total axial length of the actual contact between the magnetic fluid and shaft.

The viscous resistance torque leads to the generation of frictional dissipation power; thus, its expression can be described as follows.

(28) $$P=\Gamma \cdot \omega =4\pi \eta l\frac{\omega^{2}r_{1}^{2}r_{2}^{2}}{r_{2}^{2}-r_{1}^{2}}$$

From Equation (28), in addition to the influence of the seal’s structural parameters and rotating shaft speed, the viscosity of the magnetic fluid is an important factor affecting the dissipation power.

Based on the results and analysis in Section 2.4, the Slotte equation [24] was used to fit the viscosity–temperature characteristics of the magnetic fluid, as shown in Figure 5, and the following functional relationship was obtained.

(29) $$\eta_{H}=\frac{445.58}{{\left(T-0.94\right)}^{0.54}}$$

Substituting the above equation into Equation (9) yields the modified formula for calculating the viscous frictional dissipation power of high-speed diester-based magnetic fluid seals.

(30) $$P=\frac{1782.32}{{\left(T-0.94\right)}^{0.54}}\cdot \frac{\pi \omega^{2}r_{1}^{2}r_{2}^{2}l}{r_{2}^{2}-r_{1}^{2}}$$

## 3. Results and Discussion

### 3.1. Ignoring the Influence of Rheological Properties of Magnetic Fluid on Viscous Dissipation

The following high-speed sealing conditions were taken as an example for calculating the maximum temperature of the diester-based magnetic fluid layer. The radius of the rotating shaft was 24 mm, the rotating speed was 1000–8000 rpm, the sealing clearance was 0.1 mm, and the initial boundary temperature was 20 °C. When the rheological properties of magnetic fluids are ignored, the viscosity of the magnetic fluid can be considered as a constant under the sealing conditions, and then the heat flux is expressed as follows.

(31) $$q_{0}=\frac{\eta v_{0}^{2}}{\delta }$$

**Figure 6:**
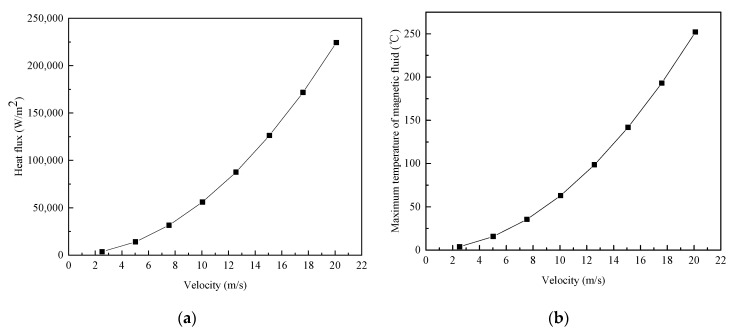
(**a**) Relationship between the linear velocity and heat flux; (**b**) maximum temperature of the magnetic fluid layer in the sealing gap at different linear velocities.

According to the above equation, the heat flux applied to the sealing gap due to viscous dissipation at different linear velocities was calculated, as depicted in Figure 6a. From the results, heat flux increases nonlinearly with angular velocity. Then, the calculation results of heat flux and other parameters were substituted into Formula (19), to obtain the calculation results of the maximum temperature of the magnetic fluid layer in seals, as shown in Figure 6b. The results show that, the higher the velocity, the higher the temperature of the magnetic fluid layer. When the temperature of magnetic fluids increases, magnetization decreases, leading to a decrease in sealing pressure resistance or even sealing failure.

**Figure 7:**
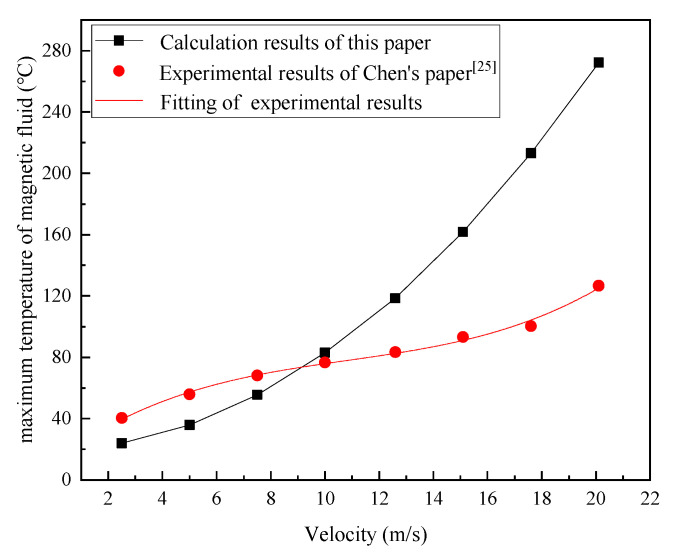
Comparison of the calculation results of magnetic fluid temperatures in the sealing gap herein, with the experimental results measured by another individual, at different linear velocities.

In order to verify the correctness of the calculation model in Section 2, the calculation results in Figure 6b were compared with the experimental results in Chen’s paper [25] (the structural parameters of the sealing test bench are the same as the sealing conditions set in this paper). As shown in Figure 7, the calculated results herein do not match very well with Chen’s measurement results. When the velocity was lower, the calculated results were smaller in value than the measured results, and the difference in values was small; when the velocity was higher, the calculated results were larger than the measured results, and the difference in values became greater. Chen measured the inner wall temperature of the pole piece near the magnetic fluid layer in the sealing gap without any cooling conditions, which is approximated as the temperature of the magnetic fluid inside the gap. Although this measurement method resulted in smaller values than the actual magnetic fluid temperature inside the sealing gap, the error was within acceptable limits. At a velocity of 10 m/s, the calculated result in this paper exhibited the best agreement with the measured result; at velocities > 10 m/s, the calculated results deviated greatly from the measured results. This indicates that the calculated results are not accurate when the velocity increases to a higher level, and the effect of the viscosity variation in the magnetic fluid on viscous dissipation cannot be ignored at this time.

### 3.2. Considering the Influence of Rheological Properties of Magnetic Fluid on Viscous Dissipation

According to the previous study, described in Section 2, temperature is the key factor affecting the viscosity of magnetic fluids under high-speed sealing conditions, and variations in viscosity affect viscous dissipation, and this affects the calculation results of the maximum temperature of magnetic fluids in the sealing gap. When sealing velocity increases, viscous dissipation increases, leading to higher temperatures and lower magnetic fluid viscosities in the sealing gap. In the above calculation process, without considering the rheological properties of magnetic fluid, the absolute viscosity at room temperature with zero magnetic field and zero shear was used, and it is larger than the viscosity at high shear rates and high temperatures, thus resulting in inaccurate maximum temperature calculations when sealing with higher speeds and exhibiting values that are larger than the actual value. Here, rheological properties were considered, and the formula for heat flux due to viscous dissipation was modified according to the viscosity–temperature function of magnetic fluids at high shear and strong magnetic field conditions; then, the formula for calculating the maximum temperature of the magnetic fluid layer in the high-speed sealing gap was corrected.

When performing viscosity calculations for diester-based magnetic fluids, based on viscosity–temperature expression (29), under the characteristics of sealing conditions, *T* in the equation denotes the average integration temperature, and it is obtained by the following equation:

(32) $$T=T_{0}+(\frac{\eta_{0}v_{0}^{2}}{3\lambda })+\frac{q_{0}\delta }{2\lambda }$$

**Figure 8:**
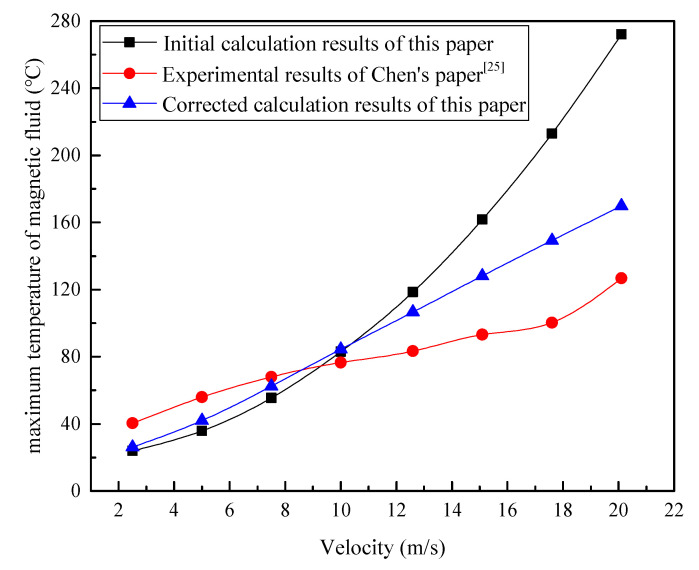
Maximum temperature of the diester-based magnetic fluid in the sealing gap at different linear velocities.

**Figure 9:**
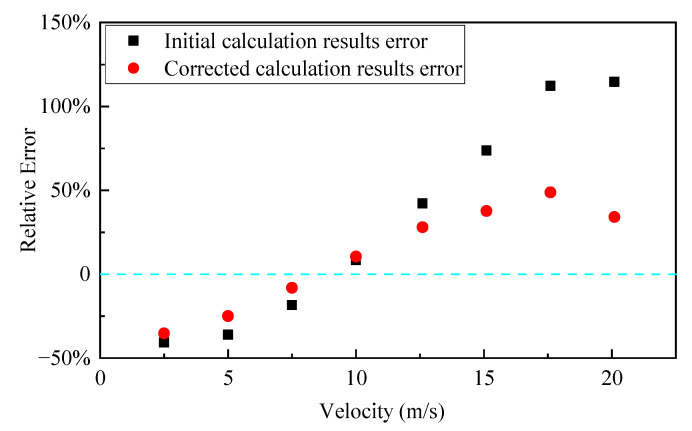
Relative error between the experimental results, and the initial calculation results and corrected calculation results.

The calculated average integration temperature at different velocities was substituted into Equation (29) to obtain the corresponding viscosity, in order to further obtain the corrected heat flux and the corrected calculation of the magnetic fluid’s maximum temperature. As shown in Figure 8, the maximum temperatures in the sealing gap at different velocities, before and after the correction, were compared with the measured results of Chen. It can be observed that the corrected calculation results at any velocity are between the initial calculation’s results and the measured results, and they are closer to the measured values than the calculation results before corrections. Therefore, this indicates that the calculation of the maximum temperature of magnetic fluids in the sealing gap, after considering the rheological properties of the magnetic fluid, is more accurate. As depicted in Figure 9, relative errors between the calculated and experimental results before and after the correction were analyzed. The error of the corrected results is smaller than the error of the initial calculated results, before the equation correction, at any velocity. In particular, the error between the calculated and measured results of maximum magnetic fluid temperatures at high velocities was significantly reduced after the calculation model was corrected. When the velocity is 20 m/s, the relative error is 34%, which is reduced by about 80% compared with the initial calculation result. According to the results shown in Figure 8, the calculated results are higher than the measured results when the velocity is ≥10 m/s, but the experimental results measured by Chen are the approximate temperatures of magnetic fluids in the sealing gap, and they are smaller than the actual values. The uncertainty in the experimental data mainly comes from the indirect measurement of the magnetic fluid’s temperature. In the magnetic fluid seal, heat is transferred radially, and the temperature gradually decreases from the shaft to the pole piece. It can be inferred that the data measured by Chen are smaller than the true temperature of the magnetic fluid. Therefore, it can be presumed that the true error of the corrected calculation results will be smaller than the error value shown in Figure 9, at higher velocities (≥10 m/s). It also means that the accuracy will be higher than the combined effect of all approximations and uncertainty. These further indicate that the calculation results when considering the rheological properties of the magnetic fluid, are more accurate at high velocities. However, when the velocity is about 18 m/s, the error is the largest, with a relative error of 48.7%. There are two possible reasons for the large error: on the one hand, the larger error in the viscosity measurement at the temperature corresponding to this velocity leads to a larger error in the calculated result; on the other hand, the larger error in the temperature measured by Chen at this velocity leads to a larger relative error. According to the comparison of experimental and simulation results in Chen’s paper [25], it is found that the agreement is also poor at this velocity. It can be inferred that the second reason is more likely, and the corrected mathematical equation for calculating the maximum temperature of the magnetic fluid layer is accurate and valid within a certain error tolerance.

As stated above, the formula for calculating the maximum temperature of the diester-based magnetic fluid layer in high-speed seals, was corrected by considering the rheological properties of the magnetic fluid as follows:

(33) $$T_{\max}=T_{0}+\frac{\eta_{H}v_{0}{}^{2}}{2\lambda }{\left(1+\frac{q_{0}\delta }{\eta_{H}v_{0}{}^{2}}\right)}^{2}$$

where $\eta_{H}$ is the shear viscosity of the magnetic fluid under the magnetic field at the average integration temperature, and $q_{0}$ is the heat flux at the corresponding viscosity, $\eta_{H}$. Via the discussion and analysis of the results in this section, we conclude that viscous dissipation, heat flux, and magnetic fluid temperatures in the seal can be calculated more accurately by considering the rheological properties of magnetic fluids, and modified Formula (33) is applicable for accurately predicting the maximum temperature of the magnetic fluid layer in the sealing gap at different velocities.

## 4. Conclusions

In the present work, a mathematical model for calculating the maximum temperature of the magnetic fluid layer in the sealing gap was established, based on the simplified high-speed sealing conditions. By analyzing the factors affecting the accuracy of the calculation model, the rheological characteristics of the magnetic fluid were measured and the viscous dissipation formula was modified. Thus, a modified calculation formula of the maximum temperature of magnetic fluids in the seals was obtained. The main conclusions from the calculation results are as follows.

Under high-speed sealing conditions, the temperature of the magnetic fluid in the sealing gap is mainly influenced by the viscous dissipation generated by viscosity, while viscosity is also influenced by the temperature. When the effect of the rheological properties of magnetic fluid on viscous dissipation is ignored, this calculation model applies to low-speed seals, and the calculation results are not accurate at high-speed seals. When the effect of the rheological properties of magnetic fluids on viscous dissipation is considered, this calculation model applies to both low- and high-speed seals, and the calculation results are more accurate. The correction formula for calculating the maximum temperature of the magnetic fluid is obtained based on the viscosity–temperature function of the magnetic fluid under high-speed sealing conditions. The correction formula applies to predicting the maximum temperature of magnetic fluid in the seal, without cooling, in the range of 0–20 m/s linear velocity. The accuracy of the temperature calculation in the higher linear velocity range needs to be further verified. Although the calculation model simplifies and ignores some influencing factors, and the calculation results have some deviations from the actual values, this calculation method is simple and convenient. Within the allowable range of error, the modified calculation model described in this paper can provide theoretical and practical reference values for quickly understanding and judging the performance of magnetic fluid seals and guiding engineering applications. In the future, we will further improve the modeling and solving methods of the temperature calculation based on this work, to obtain more accurate calculation results and wider applications.

## Figures and Tables

**Table 1:** Parameters of the examined magnetic fluid.

Samples	$\mathrm{Density}\;\rho_{ff}\;(g/{\mathrm{cm}}^{3})$	Saturation Magnetization *M*s (kA/m)	Volume Fraction of Nanoparticles φ	$\mathrm{Viscosity}\;\mathrm{at}\;\mathrm{Zero}\;\mathrm{Fields}\;\eta_{0}\;(\mathrm{mPa}.s)$	Thermal Conductivity λ(W/m·K)
Diester-based magnetic fluid	1.27	23.39	6.5%	55.5	0.178

## Data Availability

The numerical data used to support the findings of this study are included within the article.
